# Cave Actinobacteria as Producers of Bioactive Metabolites

**DOI:** 10.3389/fmicb.2019.00387

**Published:** 2019-03-22

**Authors:** Pharada Rangseekaew, Wasu Pathom-aree

**Affiliations:** ^1^Doctor of Philosophy Program in Applied Microbiology (International Program) in Faculty of Science, Chiang Mai University, Chiang Mai, Thailand; ^2^Graduate School, Chiang Mai University, Chiang Mai, Thailand; ^3^Department of Biology, Faculty of Science, Chiang Mai University, Chiang Mai, Thailand; ^4^Center of Excellence in Bioresources for Agriculture, Industry and Medicine, Chiang Mai University, Chiang Mai, Thailand

**Keywords:** actinobacteria, cave, karst, bioactive compounds, diversity, antimicrobial, anticancer, natural products

## Abstract

Recently, there is an urgent need for new drugs due to the emergence of drug resistant pathogenic microorganisms and new infectious diseases. Members of phylum Actinobacteria are promising source of bioactive compounds notably antibiotics. The search for such new compounds has shifted to extreme or underexplored environments to increase the possibility of discovery. Cave ecosystems have attracted interest of the research community because of their unique characteristics and the microbiome residing inside including actinobacteria. At the time of writing, 47 species in 30 genera of actinobacteria were reported from cave and cave related habitats. Novel and promising bioactive compounds have been isolated and characterized. This mini-review focuses on the diversity of cultivable actinobacteria in cave and cave-related environments, and their bioactive metabolites from 1999 to 2018.

## Introduction

Caves are generally regarded as any natural underground chamber that is large enough for human entrance. They can be classified based on type of rock and formation method. The most common types of caves are limestone and other calcareous rocks (Northup and Lavoie, 2001). Though caves have been studied for hundreds of years, their microbiome are generally underexplored and overlooked. Caves are attracting the interests of microbiologists, in terms of microbial diversity, during the past decade (Laiz et al., 1999; Barton et al., 2004; Barton, 2006). It is believed that microbes collected from pristine sites that are unexplored or rarely visited by humans are likely to be novel taxa or strains which produce unique beneficial chemical compounds. Market demand for new drugs is on the rise due to the emergence of new diseases and drug resistant pathogens (Genilloud, 2017; Kemung et al., 2018; Takahashi and Nakashima, 2018). With a combination of unique conditions including high humidity, relatively low and stable temperature, and low nutrients, caves are expected to harbor novel microorganisms with biotechnological benefits. Members of actinobacteria are reported to be a dominant microbial population in several cave ecosystems (Groth and Saiz-Jimenez, 1999; Cheeptham et al., 2013; Tomczyk-Zak and Zielenkiewicz, 2016; Ghosh et al., 2017).

Actinobacteria are large group of high G+C Gram positive bacteria (Barka et al., 2016). They are regarded as the most prolific source of bioactive compounds in particular commercially available antibiotics. Actinobacteria produce approximately two-thirds of all know antibiotics in the market, most of these are from members of the genus *Streptomyces* (Barka et al., 2016). Several members of diverse actinobacterial taxa were also found to produce wide range of other biologically active compounds, for examples antibacterial, anticancer, or antifungal drugs (Barka et al., 2016; Genilloud, 2017; Castro et al., 2018; Takahashi and Nakashima, 2018). Isolation of actinobacteria from unique natural habitats is of interest to avoid re-isolation of strains that produce known bioactive metabolites and usually lead to highly diverse actinobacterial communities. The present mini-review provides evidence that actinobacteria from caves are expected to be a good source for drug discovery (Yücel and Yamac, 2010; Cheeptham et al., 2013; Kay et al., 2013; Ghosh et al., 2017; Riquelme et al., 2017).

## Selective Isolation of Cave Actinobacteria

In the past decade, there are many reports on the discovery of novel actinobacteria in cave habitats. Successful isolation of actinobacteria from caves depend largely on factors of (1) media composition (Kim et al., 1998) (2) culture condition, and (3) pretreatment methods (Kim et al., 1998; Nakaew et al., 2009a,b; Duangmal et al., 2012; Niyomvong et al., 2012; Velikonja et al., 2014; Fang et al., 2017b; Adam et al., 2018). Media used for the isolation of cave actinobacteria range from routine cultivation media such as International *Streptomyces* Project medium 2 (yeast malt extract agar, ISP2) or tryptic soy agar (TSA) to selective media including humic acid vitamin agar (HV), starch casein agar (SC), starch casein nitrate agar (SCN), peptone-yeast extract/brain-heart infusion medium (PY-BHI), R2A medium, actinomycete isolation agar (AI), and Gauze's medium No.1. Moreover, isolation media that mimic the conditions of low concentration nutrients in caves such as tap water agar, 1/100 ISP2 and oligotrophic medium (M5) were also successfully used for the isolation of actinobacteria. (Lee et al., 2000b; Velikonja et al., 2014; Covington et al., 2018; Passari et al., 2018). High concentration of nutrients in standard cultivation media were reported to cause cell death in cave-associated bacteria due to osmotic stress (Barton, 2006; Ghosh et al., 2017).

Two important culture conditions for actinobacteria isolation are incubation temperature and incubation time. Four incubation temperatures (5°, 13°, 20°, and 28°C) were used for the isolation of soil bacteria including actinobacteria from three caves in Northern Spain (Laiz et al., 2003). The incubation temperature of 5°C was used to represent cave temperature and target psychrotrophs, 28°C as laboratory incubation temperature and 20°C as intermediate temperature between cave and laboratory conditions. The highest number of actinobacterial isolates (mostly sporoactinomycetes) was obtained at 28°C followed by 13°, 20° and 5°C, respectively. However, a higher diversity was observed from 13°C than 28°C. Therefore, these authors concluded that the isolation of actinobacteria is a temperature-dependent process. In addition, longer incubation time was successfully used to promote the recovery of slow-growing actinobacteria (Laiz et al., 2003).

Pretreatment, both chemical and physical methods are generally useful for isolation of various actinobacterial species. Physical pretreatments involve the use of air drying, moist heat, dry heat and electromagnetic wave. Moist heating (water bath at 50°C for 5–6 min) is useful for eliminating of fast growing bacteria (Niyomvong et al., 2012; Velikonja et al., 2014). Dry heating at 120°C for 1 h is effective in reducing number of unwanted bacteria and found to be an effective method for isolation of members of the genera *Dactylosporangium, Streptosporangium* and *Microbispora*, while growth of streptomycetes was limited (Jiang et al., 2016). In addition, dry heating with or without phenol treatment resulted in a reduction of bacteria and heat-labile *Streptomyces*, thus heat resistant rare actinobacteria were readily isolated (Kim et al., 1998; Nakaew et al., 2009a; Niyomvong et al., 2012). However, these treatments also affect the number of viable actinobacteria (Niyomvong et al., 2012). Pretreatment using microwave irradiation was effective for the isolation of rare actinobacteria (Niyomvong et al., 2012) and capable of inducing spore germination in some species of *Streptomyces, Nocardia, Streptosporangium, Lentzea, Micromonospora*, and *Micropolyspora* (currently transferred to *Nocardia*) (Bulina et al., 1997; Wang et al., 2013; Velikonja et al., 2014). For chemical pretreatment, the type and concentration of calcium salts are important for the isolation of actinobacteria (Fang et al., 2017b). Selective media supplemented with CaCO_3_ yield higher actinobacterial count than those supplemented with CaCl_2_ and (CH_3_COO)_2_Ca. The concentration of these three salts, at low concentration (0.1 and 0.01% (w/v) yield higher CFU of actinobacteria than in its absence or at high concentration. Calcium is important for environmental stress tolerance in actinobacteria because calcium forms a compound with dipicolinic acid as calcium dipicolinate and acts as secondary stabilizing agent for spore against environmental stress (Moir and Smith, 1990).

## Novel Actinobacterial Taxa

Several novel actinobacterial taxa isolated from caves and cave related habitats during the period of 20 years from 1999 to 2018 were summarized in Table 1. In total, 47 species within 30 genera were described including 7 novel genera. The highest number of novel species was from genus *Streptomyces* (5) followed by *Amycolatopsis* (4) and *Nocardia* (4). The majority of these novel actinobacteria were isolated from cave soils including 6 novel genera, *Antricoccus, Beutenbergia, Knoellia, Lysinibacter Spelaeicoccus* and *Sphaerimonospora*. Only the genus *Hoyosella* was recovered from complex biofilm on the ceiling and wall of Altamira cave, Spain. The extreme conditions within the caves are expected to create stress for the inhabitant microorganisms at the genetic level, paving the way for the evolution of new species and their novel metabolites (Tawari and Gupta, 2013). Therefore, caves are considered as an attractive source for the isolation of novel actinobacterial taxa.

**Table 1:** Novel actinobacterial taxa isolated from cave and related habitats between 1999 and 2018.

**Family**	**Genus**	**Species**	**Sources**	**Media**	**References**
*Brevibacteriaceae*	*Spelaeicoccus*	*Spelaeicoccus albus*	Soil from natural cave in Jeju, Korea	Starch casein agar	Lee, 2013b
*Conexibacteraceae*	*Conexibacter*	*Conexibacter stalactiti*	Pieces of stalactites from Yongcheon cave in Jeju, Korea	Starch casein agar	Lee, 2017
*Glycomycetaceae*	*Stackebrandtia*	*Stackebrandtia cavernae*	Rocks from karst cave, Guizhou, south-west China	R2A agar with 0.1% CaCO_3_	Zhang et al., 2016
*Intrasporangiaceae*	*Fodinibacter*	*Fodinibacter luteus*	Sample from wall of a salt mine in Yunnan, China	Marine agar 2216	Wang et al., 2009
	*Knoellia*	*Knoellia sinensis*	Soils from the Reed Flute cave near Guilin, Guangxi, China	Casein mineral medium	Groth et al., 2002
		*Knoellia subterranea*	Soils from the Reed Flute cave near Guilin, Guangxi, China	Peptone/yeast extract/brain- heart infusion medium (PY-BHI)	Groth et al., 2002
	*Ornithinimicrobium*	*Ornithinimicrobium cavernae*	Stalagmites from karst cave in Luoyang country, Henan, northern China	ISP 2 with nystatin and nalidixic acid	Zhang et al., 2018
*Kineosporiaceae*	*Augustibacter*	*Augustibacter spluncae*	Pieces of stalactites from Yongcheon cave in Jeju, Korea	Starch casein agar	Ko and Lee, 2017
*Microbacteriaceae*	*Agromyces*	*Agromyces subbeticus*	Cyanobacterial biofilm from Cave of Bats, near Zuheros, Cordoba, southern Spain	Peptone/yeast extract/ brain- heart infusion medium (PY-BHI)	Jurado et al., 2005
	*Humibacter*	*Humibacter antri*	Clay soils from natural cave in Jeju, Korea	Starch casein agar	Lee, 2013a
	*Lysinibacter*	*Lysinibacter cavernae*	Soils from wild karst cave in the Wulong region, Chongqing, China	FA (fulvic acid) agar	Tuo et al., 2015
*Micrococcaceae*	*Arthrobacter*	*Arthrobacter psychrophenolicus*	Carbonate-rich deposit from Alpine ice cave, Salzburg, Austria	Soil-extract agar	Margesin et al., 2004
	*Beutenbergia*	*Beutenbergia cavernae*	Soils from the Reed Flute cave near Guilin, Guangxi, China	Casein mineral medium and peptone/yeast extract/brain-heart infusion medium	Groth et al., 1999
*Micromonosporaceae*	*Catellatospora*	*Catellatospora koreensis*	Soils from gold-mine cave in Kongju, Korea	Yeast extract; glucose; K_2_HPO_4_;Na_2_HPO_4_;KNO_3_; NaCl; MgSO_4_.7H_2_O; CaCl_2_.2H_2_O and trace mineral solution	Lee et al., 2000a
	*Micromonospora*	*Micromonospora kangleipakensisi*	Sample from limestone quarry at Hundung, Manipur, India	Gauze's medium	Nimaichand et al., 2013c
*Mycobacteriaceae*	*Hoyosella*	*Hoyosella altamirensis*	Complex bioflim on the cave ceiling and walls from Altamira cave, Cantabria, Spain	Starch casein agar	Jurado et al., 2009
*Nocardioidaceae*	*Jiangella*	*Jiangella alkaliphila*	Soils from natural cave on Jeju island, Korea	Starch casein agar	Lee, 2008
	*Nocardioides*	*Nocardioides cavernae*	Soils from karst cave in Xingyi county, Guizhou, south-western China	R2A agar with cycloheximide and nalidixic acid	Han et al., 2017
	*Tenggerimyces*	*Tenggerimyces flavus*	Soil from Shenxian cave, Henan, China	R2A agar with cycloheximide, nalidixic acid and potassium dichromate	Li et al., 2016
*Norcardiaceae*	*Nocardia*	*Nocardia altamirensis*	Complex microbial community forming a gray-colored colonization on the walls from Altamira cave, Cantabria, Spain	Tryptose soy agar	Jurado et al., 2008
		*Nocardia cavernae*	Soil from karst cave in Xingyi county, Guizhou, south-western China	Humic acid-vitamin agar with cycloheximide and nalidixic acid	Li et al., 2017
		*Nocardia jejuensis*	Soil from natural cave on Jeju island, Korea	Starch casein agar	Lee, 2006c
		*Nocardia speluncae*	Soil from natural cave on Jeju island, Korea	Starch casein agar	Seo et al., 2007
	*Rhodococcu*s	*Rhodococcus antrifimi*	Dried bat dung from natural cave in Jeju, Korea	Starch casein agar	Ko et al., 2015
		*Rhodococcus canchipurensis*	Soil from limestone quarry at Hundung, Manipur, India	Starch casein nitrate agar	Nimaichand et al., 2013a
*Propionibacteriaceae*	*Microlunatus*	*Microlunatus cavernae*	Soil from Alu ancient cave, Yunnan, south-west China	R2A medium	Cheng et al., 2013a
*Pseudonocardiaceae*	*Amycolatopsis*	*Amycolatopsis halotolerans*	Soil from natural cave in Jeju island, Korea	Starch/casein agar	Lee, 2006b
		*Amycolatopsis jejuensis*	Dried bat dung from natural cave in Jeju island, Korea	Starch casein agar	Lee, 2006b
		*Amycolatopsis jiguanensis*	Soil from Ji Guan cave, Henan, middle-eastern China	ISP2	Huang et al., 2016
		*Amycolatopsis xuchangensis*	Soil from Ji Guan cave, Henan, middle-eastern China	ISP2	Huang et al., 2016
	*Lentzea*	*Lentzea cavernae*	Limestone from karst cave in Xingyi county, Guizhou, south-western China	Humic acid-vitamin agar with cycloheximide and nalidixic acid	Fang et al., 2017a
		*Lentzea guizhouensis*	Limestone from Puding karst ecosystem research station of the Chinese Academy of Sciences in Guizhou Province, south-west china	Modified ATCC-172 medium at 1/10 concentration	Cao et al., 2015
	*Saccharothrix*	*Saccharothrix albidocapillata*	Soil from gold mine cave in Kongju, Korea	Tap water agar and oligotrophic (M5) medium	Lee et al., 2000b
		*Saccharothrix violacea*	Soil from gold mine cave in Kongju, Korea	Tap water agar and oligotrophic (M5) medium	Lee et al., 2000b
	*Saccharopolyspora*	*Saccharopolyspora cavernae*	Swallow cave, Yunnan, south-west China	Improved DSMZ medium 405	Cheng et al., 2013b
*Streptomycataceae*	*Streptomyces*	*Streptomyces boninensis*	Soil from limestone cave, Ogasawa islands, Tokyo, Japan	Humic acid-vitamin agar with benlate and nalidixic acid	Také et al., 2018
		*Streptomyces canchipurensis*	Soil from limestone quarry at Hundung, Manipur, India	Gauze's medium No. 1	Li et al., 2014
		*Streptomyces hundungensis*	Soil from limestone quarry at Hundung, Manipur, India	Starch casein nitrate agar	Nimaichand et al., 2013b
		*Streptomyces lunaelactis*	Moonmilk deposit from Grotte des Collemboles cave in Comblain-au-Pont, Belgium	ISP and starch nitrate (SN) medium with nalidixic acid and nystatin	Maciejewska et al., 2015
		*Streptomyces manipurensis*	Soil from limestone quarry at Hundung, Manipur, India	Starch casein Nitrate agar	Nimaichand et al., 2012
*Streptosporangiaceae*	*Sphaerimonospora*	*Sphaerimonospora thailandensis* (formerly *Microbispora thailandensis*)	Soil from tropical limestone cave (Khao No-Khao Kaeo), Nakorn Sawan, Thailand	Humic acid-vitamin agar with nalidixic acid and ketoconazole	Duangmal et al., 2012
		*Sphaerimonospora cavernae*	Soil from tropical limestone cave (Khao No-Khao Kaeo), Nakorn Sawan, Thailand	-	Mingma et al., 2016
	*Nonomuraea*	*Nonomuraea monospora*	Soil from Pha Tup cave forest park, Nan, Thailand	Humic acid-vitamin agar with nystatin and cycloheximide	Nakaew et al., 2012
		*Nonomuraea indica*	Soil from limestone open pit mine from Gulbarga region, Karnataka, India	Starch casein agar	Quadri et al., 2015
*Thermomonosporaceae*	*Actinocorallia*	*Actinocorallia cavernae*	Natural cave in Jeju, Korea	Starch/casein agar	Lee, 2006a
Not assigned to family (Suborder *Frankineae*)	*Antricoccus*	*Antricoccus suffuscus*	Soil from natural cave in Jeju, Korea	Starch casein agar	Lee, 2015

Most species were isolated from selective media that were designed for the isolation of actinobacteria such as humic acid vitamin agar, starch casein agar, starch casein nitrate agar. However, some novel species were isolated using general cultivation media such as ISP2 media (*Amycolatopsis jiguanensis* and *A. xuchangensis*) and TSA (*Nocardia altamirensis*). In addition, low nutrient media (tap water agar and oligotrophic M5 media) were preferable for the isolation of *Saccharothrix violacea* and *S. albidocapillata*. Most novel species were incubated at 28°–30°C for 1–6 weeks. However, *Arthrobacter psychrophenolicus* was isolated at 4°C, this may be because this species originated from Alpine ice cave in Salzburg, Austria (Margesin et al., 2004). *Lysinibacter cavernae* was isolated at 15°C from soil in a wild karst cave in the Wulong region, Chongqing, China (Tuo et al., 2015). *Streptomyces lunaelactis* was isolated at 17°C from a moonmilk deposit in the Grotte des Collemboles cave in Belgium (Maciejewska et al., 2015).

Pretreatment procedures were also useful for isolation of some novel species. For example, *Microbispora thailandensis* was isolated from soil pretreated with microwave radiation at a frequency of 2460 MHz and power setting of 100 W for 45 s (Duangmal et al., 2012). *Nonomuraea monospora* was isolated from soil treated with phenol (Nakaew et al., 2012). *Streptomyces manipurensis* was isolated from soil supplemented with 0.1 g of CaCO_3_ for 1 day to prevent the growth of fast growing bacteria (Nimaichand et al., 2012).

## Bioactive Compounds From Cave Actinobacteria

Caves are extreme habitats with low nutrient, temperature and light intensity but have high humidity (Schabereiter-Gurtner et al., 2002). These unique characteristics may promote the production of bioactive substances in particular antibiotics by actinobacteria (Nakaew et al., 2009a). Bioactive metabolites from cave associated actinobacteria have been purified, their structure elucidated and reported in recent years (Table 2). These compounds mostly displayed anti-bacterial and/or anti-cancer activities. The most prolific producer is members of the genus *Streptomyces*.

**Table 2:** Bioactive metabolites from cave actinobacteria.

**Bioactivity**	**Compounds**	**Structure**	**Producing strain**	**Source of strain**	**References**
Antibacterial	Cervimycins A, B, C, and D	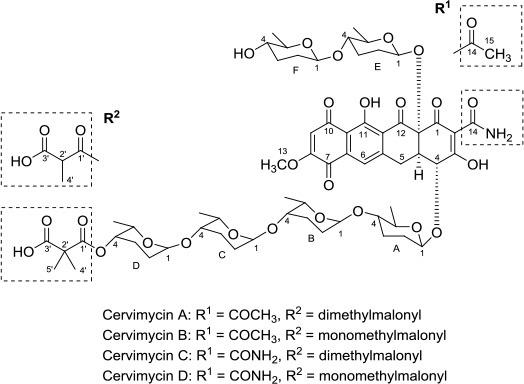	*Streptomyces tendae* strain HKI 0179	Rock wall from Ancient cave, The Grotta dei Cervi, Italy	Herold et al., 2005
	Undecylprodigiosin	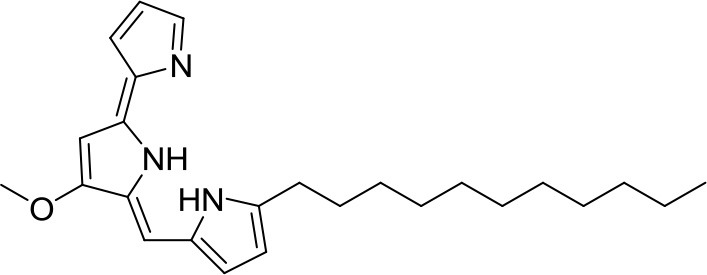	*Streptomyces* sp. JS520	Cave on mountain Miroc, Serbia	Stankovic et al., 2012
	Xiakemycin A	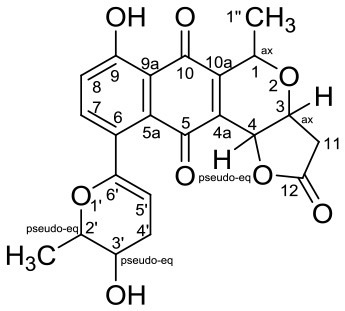	*Streptomyces* sp. CC8-201	Soil from karst cave,Chongqing city, China	Jiang et al., 2015
	Chaxalactin B	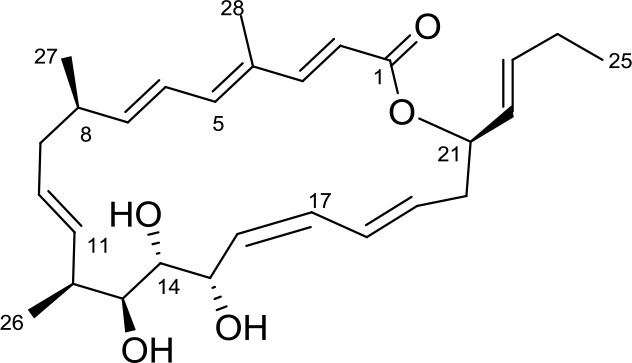	*Streptomyces* sp. IB 2014/I/ 78-8	Bolshaya Oreshnaya cave in the Mansk area of the Krasnoyaesk, Siberia, Russia	Axenov-Gibanov et al., 2016
Anticancer	Hypogeamicins A	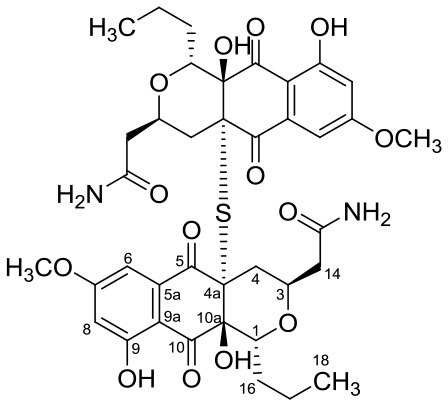	*Nonomuraea specus*	Hardin's cave system located close to Ashland City, Tennessee	Derewacz et al., 2014
	Xiakemycin A	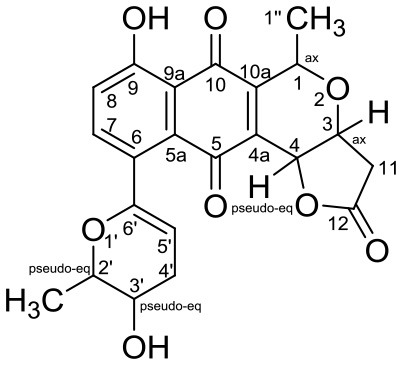	*Streptomyces* sp. CC8-201	Soil from karst cave,Chongqing city, China	Jiang et al., 2015
	Huanglongmycin (HLM) A,	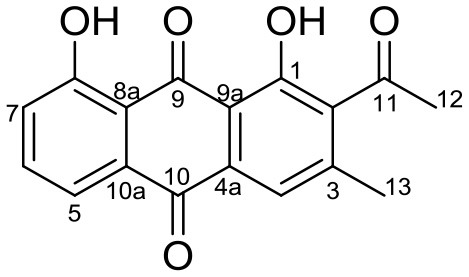	*Streptomyces* sp. CB09001	Soil from karstic cave in Xiangxi, China	Jiang et al., 2018
Antioxidative activity	Undecylprodigiosin	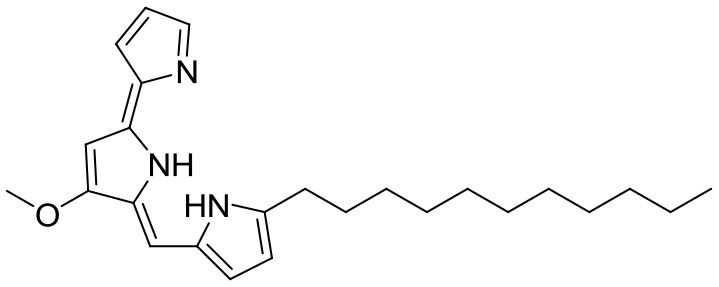	*Streptomyces* sp. JS520	Cave on mountain Miroc, Serbia	Stankovic et al., 2012
Inhibitory activity against lipid metabolism	Gyrophoric acid	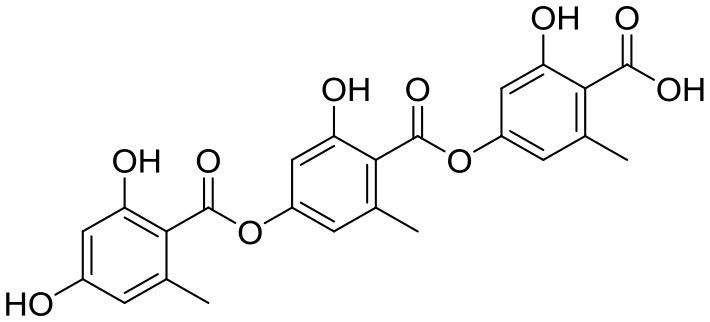	*Streptomyces* sp. IB 2014/I/ 78-8	Bolshaya Oreshnaya cave in the Mansk area of the Krasnoyaesk, Siberia, Russia	Axenov-Gibanov et al., 2016 Tomoda and Omura, 2001

Cervimycin A, B, C, and D were produced from *Streptomyces tendae* strain HKI 0179, isolated from a rock wall in an ancient cave, the Grotta dei Cervi in Italy. Cervimycins A and B are novel polyketide glycosides. However, cervimycin C and D have the same structure as known compounds A2121-3 and A2121-2. Cervimycins A-D are highly active against Gram positive bacteria (*B. subtilis* and *S. aureus*) and multi-drug-resistant *S. aureus* (MRSA), vancomycin-resistant *Enterococcus faecalis* (VRE) and efflux-resistant *S. aureus* EfS4 (Herold et al., 2005).

Xiakemycin A is a novel pyranonaphthoquinone (PNQ) antibiotics produced by *Streptomyces* sp. CC8-201 from remote karst soil in China. Xiakemycin A showed strong inhibitory activities against Gram positive bacteria (*S. aureus, S. epidermidis, E. faecalis, and E. faecium*) and cytotoxic against numerous cancer cell lines (human lung cancer A549 cells, breast cancer MCF-7 cells, hepatoma HepG-2 cell, cervical cancer HeLa cells, colon carcinoma HCT-116 cell p53 wt cells, neuroblastoma SH-SY5Y cells, and human prostate cancer PC-3) (Jiang et al., 2015).

Hypogeamicins A, B, C, and D were produced by *Nonomuraea specus* isolated from Hardin's cave system in Tennessee, USA. Hypogeamicin A showed cytotoxicity to colon cancer cell line TCT-1 while hypogeamicin B-D were active against *B. subtilis* with no cytotoxicity to TCT-1. However, hypogeamicin B-D are not as potent as erythromycin and gentamicin in terms of antimicrobial activity against *B. subtilis* (Derewacz et al., 2014).

Huanglongmycin A, B, and C are aromatic polyketides from *Streptomyces* sp. CB09001, isolated from karstic cave soil of Xiangxi, China. Huanglongmycin A showed a weak anti-Gram negative bacteria (*Pseudomonas aeruginosa* and *Escherichia coli*) and moderate cytotoxicity against A549 lung cancer cell line. Huanglongmycin B has weak antibacterial activity against *S. aureus* and multi-drug-resistant *S. aureus* (MRSA). Huanglongmycin C showed neither antibacterial nor anticancer activities (Jiang et al., 2018). Undecylprodigiosin was produced by *Streptomyces* sp. JS520 isolated from sediments in cave in the mountain Miroc, Serbia. Undecylprodigiosin is a deep red pigment with antibacterial activity against *Micrococcus luteus, B. subtilis*, and *C. albicans*. Moreover, undecylprodigiosin also showed antioxidative and UV-protective properties (Stankovic et al., 2012).

Four known compounds with bioactivity (cyclodysidin D, chaxalactin B, stylissazole B, and gyrophoric acid) were reported to produce by *Streptomyces* sp. IB 2014/I/ 78-8 from moonmilk speleothem of Bolshaya Oreshnaya cave in Siberia (Axenov-Gibanov et al., 2016). Cyclodysidin D is previously reported in marine sponge, *Dysidea tupha* associated *Streptomyces* sp. RV 15. This compound showed no activity against bacteria, fungi and parasites (Abdelmohsen et al., 2014). Chaxalactin B was produced from *Streptomyces* sp. C34 from a hyper-arid soil samples collected from the Atacama Desert, Chile. This compound has strong activity against Gram positive bacteria (Castro et al., 2018). Stylissazole B was isolated from the marine sponge *Stylissa carteri* collected in the Solomon islands but no report on bioactivity (Patel et al., 2010). Gyrophoric acid isolated from *Humicola* sp. FO-2942 is an inhibitor of diacylglycerol acyltransferase and a lipid-lowering agent (Inokoshi et al., 2010).

## Bioactivity of Uncharacterized Compounds

Several cave actinobacteria have been screened for their biological activity such as antibacterial, anticancer and antifungal. However, no pure compound and their structure were reported in these studies. The screening of only bioactivity without the structure elucidation of bioactive metabolites may not useful for the discovery of new antibiotics (Hug et al., 2018). Nevertheless, these findings provide evidence which supports the potential of cave actinobacteria to be exploited for novel bioactive compounds.

Turkish karstic caves were reported to harbor actinobacteria, for which 62% of the isolates, were active against several microbial pathogens (Gram positive bacteria, Gram negative bacteria, yeast, and filamentous fungi). *Streptomyces* sp. 1492 had strong activity against clinical strains of MRSA, VRE, and *Acinetobacter baumanii* (Yücel and Yamac, 2010). *Streptomyces* E9 isolated from Helmcken Falls cave in British Columbia could inhibit the growth of *Paenibacillus larvae*, a causative agent of American foulbrood disease in honeybees (Kay et al., 2013). A moonmilk speleothems of limestone caves, Grotte des Collemboles in Belgium were investigated for antimicrobial producing cultivable actinobacteria. A collection of obtained *Streptomyces* displayed strong inhibitory activity against Gram positive and Gram negative bacteria (Maciejewska et al., 2016). In a study of cultivable actinobacteria from Azores volcanic caves in Portugal, 18.1% of 148 actinobacterial isolates have antibacterial activity against at least one of the following bacteria: *Salmonella typhimurium, E. coli, P. aeruginosa, Proteus* sp., *Listeria monocytogenes, L. innocua*, and *S. aureus*. Most of the active isolates belong to the genus *Streptomyces* (*S*. *nojiriensis, S*. *spiroverticillatus, S*. *avidinii*, and *S*. *mauvecolor*) followed by *Arthrobacter* (Riquelme et al., 2017). A total of 40 taxa belonging to the genera *Agromyces, Amycolatopsis, Kocuria, Micrococcus, Micromonospora, Nocardia, Streptomyces*, and *Rhodococcus* were recovered from moonmilk deposits inside the Grotte des Collemboles, Belgium. Antimicrobial activity was found in isolated strains against Gram positive bacteria (87%) and Gram negative bacteria (59%) (Adam et al., 2018). Sixteen isolates of *Streptomyces* spp. from Chaabe cave in Algeria were screened for their antimicrobial activity using agar cylinder method. All of them showed strong anti-Gram positive (*S. aureus, M. luteus, L. monocytogenes*, and *B. subtilis)* activity (Belyagoubi et al., 2018).

For anticancer activity, a rare actinobacterium *Spirillospora albida* strain CMU-PNK470 was isolated from Phanangkhoi cave in northern Thailand (Nakaew et al., 2009a). This bacterium showed activity against human small lung cancer cell (NCI-H1870) with an IC_50_ value of 10.18 μg/ml. Similarly, *Nonomurea roseola* strain PT708 isolated from Phatup cave forest park in northern Thailand was tested positive for anticancer activity against human oral cavity cancer (KB) and human small lung cancer cells (NCI-H187) (Nakaew et al., 2009b). Moreover, these two strains are also active against some Gram positive pathogenic bacteria (*B. cereus*, MRSA, and *Paenibacillus larvae*).

Some examples of antifungal activity from cave actinobacteria have been reported. Antagonistic *Streptomyces, Micromonospora, Streptosporangium*, and *Dactylosporangium* were isolated from five caves (Cheondong, Kosoo, Nadong, Seonglyu, and Ssangyong) in Korea (Kim et al., 1998). They showed activity against at least one of plant pathogenic fungi (*Alternaria solani, Colletotrichum gloeosporioides, Fusarium oxysporum* f.sp. *lycopersici, Magnaporthe grisea, Phytophthora capsici*, and *Rhizoctonia solani*). Similarly, members of genera *Streptomyces* and *Janibacter* isolated from limestone deposit sites in Hundung, Manipur, India were reported to show anticandidal and biocontrol activities against rice fungal pathogens (*Curvularia oryzae, F. oxysporum, Helminthosporum oryzae, Pyricularia oryzae, R. pryzae-sativae*, and *R. solani*) as well as antibacterial activity (Nimaichand et al., 2015). However, *Amycolatopsis, Rhodococcus*, and *Pseudonocardia* isolates showed only biocontrol activity against rice fungal pathogen. Recently, five *Streptomyces* spp. from Chaabe cave in Algeria was reported to produce non-polyenic antifungal substances active against *C. albicans* (Belyagoubi et al., 2018).

## Conclusion and Future Perspectives

Emerging and re-emerging infectious diseases are threatening human society at an alarming rate. It is a call of emergency to find an effective cure for these pathogens. Actinobacteria are proving again to be prolific producers of promising bioactive compounds with widely application. Cave and karst environments are underexplored microbiologically and should not be overlooked for the search and discovery of novel actinobacteria and their chemical diversity of useful compounds. It is evident from this mini-review that cave environments harbor novel and diverse actinobacteria (Table 1). These actinobacteria offer a rich source of bioactive compounds as exemplified in Table 2. We opine that in order to explore cave actinobacteria to their full potential, 2 major research area must be addressed. The first area of research should deal with the ability to isolate and cultivate actinobacteria of interest. It is well-accepted that most microorganisms could not be cultivated in laboratory. The isolation and cultivation of bioactive producing actinobacteria under laboratory conditions represent the first challenge. Currently, the isolation strategy specifically for cave actinobacteria is lacking. There is still an urgent need for an improved selective isolation to target specific actinobacterial taxa of interest and extended our ability to tap into the majority of these uncultivable bacteria. Modification of growth conditions and use of new culturing methods were proposed for cultivation of previously uncultivable microorganisms (Pham and Kim, 2012). A combination of enrichment techniques including heat-pretreatments of samples, adjusting media pH and calcium salts supplements were effectively applied to isolate rare actinobacteria from karstic caves (Fang et al., 2017b).

The advancement of next generation sequencing and accumulation of high quality whole genome data provide a powerful tool and useful information to support the search for novel bioactive metabolites for drug development. Currently, these genome data of actinobacteria reveal the presence of several biosynthetic gene clusters of secondary metabolites and reaffirm status of actinobacteria as prolific producers of bioactive compounds. However, these gene clusters are not normally expressed under laboratory conditions. Many secondary metabolites encoded by these gene clusters remain unidentified in fermentation broth (Scherlach and Hertweck, 2009; Ren et al., 2017). Therefore, the second challenge lies in our ability to activate these silent gene clusters. Recently, specific biological and chemical stimuli namely exposure to antibiotics, metals and mixed microbial culture, were successfully employed to activate secondary metabolites production in cave actinobacteria (Covington et al., 2018). Evidently, the study on cave actinobacteria and their bioactive compounds is still at an early stage. There still remains room for further study to guarantee cave actinobacteria as producers of new bioactive compounds for the benefit of human well-being.

## Author Contributions

PR contributed data for selective isolation, novel taxa, bioactive metabolites and Tables 1, 2. WP conceived the idea, wrote, and revised the whole manuscript.

### Conflict of Interest Statement

The authors declare that the research was conducted in the absence of any commercial or financial relationships that could be construed as a potential conflict of interest.
